# New Insights Into the Epigenetic Regulation of Inflammatory Bowel Disease

**DOI:** 10.3389/fphar.2022.813659

**Published:** 2022-01-31

**Authors:** Jing Xu, Hao-ming Xu, Mei-feng Yang, Yu-jie Liang, Quan-zhou Peng, Yuan Zhang, Cheng-mei Tian, Li-sheng Wang, Jun Yao, Yu-qiang Nie, De-feng Li

**Affiliations:** ^1^ Department of Gastroenterology and Hepatology, Guangzhou Digestive Disease Center, Guangzhou First People’s Hospital, School of Medicine, South China University of Technology, Guangzhou, China; ^2^ Department of Hematology, Yantian District People’s Hospital, Shenzhen, China; ^3^ Shenzhen Kangning Hospital, Shenzhen, China; ^4^ Department of Pathology, Shenzhen People’s Hospital (The Second Clinical Medical College, Jinan University, The First Affiliated Hospital, Southern University of Science and Technology), Shenzhen, China; ^5^ Department of Medical Administration, Huizhou Institute of Occupational Diseases Control and Prevention, Huizhou, China; ^6^ Department of Emergency, Shenzhen People’s Hospital (The Second Clinical Medical College, Jinan University, The First Affiliated Hospital, Southern University of Science and Technology), Shenzhen, China; ^7^ Department of Gastroenterology, Shenzhen People’s Hospital (The Second Clinical Medical College, Jinan University, The First Affiliated Hospital, Southern University of Science and Technology), Shenzhen, China

**Keywords:** inflammatory bowel disease, epigenetics, inflammatory, DNA methylation, histone modifications, miRNA

## Abstract

Inflammatory bowel disease (IBD) is a chronic inflammatory disease of the colonic mucosa. Environmental factors, genetics, intestinal microbiota, and the immune system are all involved in the pathophysiology of IBD. Lately, accumulating evidence has shown that abnormal epigenetic changes in DNA methylation, histone markers, and non-coding RNA expression greatly contribute to the development of the entire disease. Epigenetics regulates many functions, such as maintaining the homeostasis of the intestinal epithelium and regulating the immune system of the immune cells. In the present study, we systematically summarized the latest advances in epigenetic modification of IBD and how epigenetics reveals new mechanisms of IBD. Our present review provided new insights into the pathophysiology of IBD. Moreover, exploring the patterns of DNA methylation and histone modification through epigenetics can not only be used as biomarkers of IBD but also as a new target for therapeutic intervention in IBD patients.

## Introduction

As a chronic inflammatory disease, inflammatory bowel disease (IBD) is characterized by recurrent abdominal pain and diarrhea, and its subtypes include ulcerative colitis (UC) and Crohn’s disease (CD). The incidence of IBD has been increasing day by day, especially in developing countries undergoing industrialization and urbanization, which brings a great burden to the country and society (Burisch and Munkholm, 2015). IBD is a heterogeneous disease, and its pathogenesis remains largely unknown. However, it is considered to be mainly related to heredity, environment, immunity, and gut microbiota.

**FIGURE 1:**
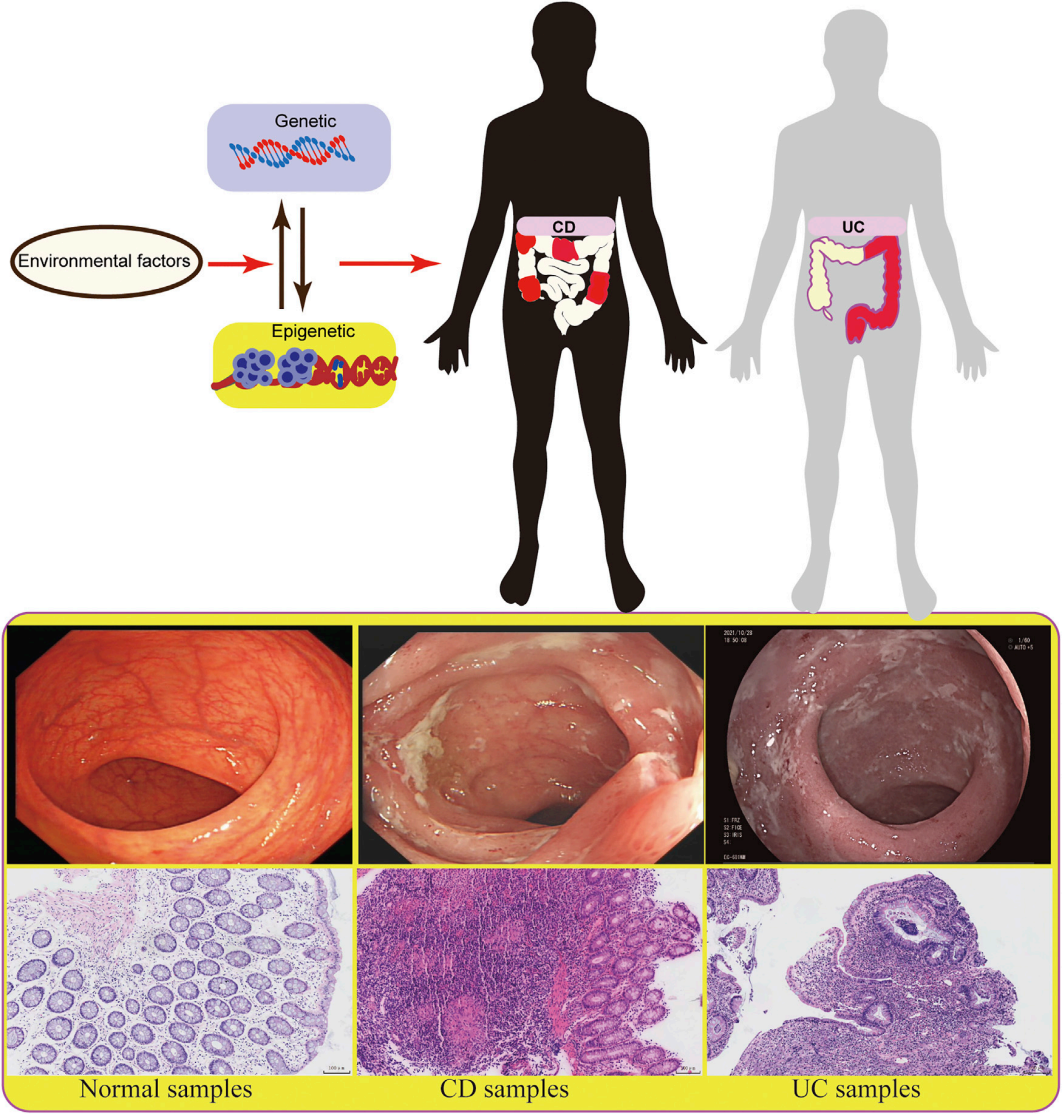
The diagram shows different factors of epigenetics in IBD.

Studies on genetic factors have revealed some susceptibility gene loci related to IBD, while they are not decisive factors for the etiology, complexity, and evolution of IBD (Jostins et al., 2012; Gordon et al., 2015). Environmental factors, especially epigenetic factors, may participate in the occurrence and development of IBD and play an important role in the research of disease pathogenesis (Chen et al., 2017; Treviño et al., 2020). About 70% of IBD risk sites are the same as other autoimmune diseases, such as psoriasis and rheumatoid arthritis, indicating that genetic factors provide limited indication (Zhernakova et al., 2009). The role of non-genetic factors in IBD has been well proved. As two major phenotypes of IBD, UC and CD have different clinical phenotypes, endoscopic manifestations, and pathological features. Most studies have shown that these two subtypes of IBD can be developed under the same genetic, environmental, and intestinal flora conditions, highlighting the contribution of epigenetic factors to IBD (Goodrich et al., 2014). Besides, the specific phenotype may be related to some special environments. However, the underlying specific mechanism remains to be explored (Figure 1). Epigenetics refers to the regulation of gene expression without changing genetic information. Because of its heritability, reversibility and dynamics, epigenetics is more beneficial to participate in the development, differentiation, and function of the host. In addition, the correlation between epigenetics and IBD is helpful for early diagnosis and disease classification of patients, providing a new research direction and treatment choice for IBD. In the present work, to emphasize the importance of epigenetic mechanisms for IBD, we reviewed the effects of DNA methylation, histone modification, non-coding RNA (ncRNA), and epigenetic modification on T and B immune cells in IBD.

### The Epigenetic Mechanism of IBD

Epigenetics refers to the mechanism of regulating gene expression without changing the DNA sequence, and its process mainly includes DNA methylation, histone modification, ncRNA and chromatin interaction. Epigenetics is affected by the environment, and the changes such as DNA methylation, can be stably inherited between cell generations. An epigenetic mechanism has various regulatory modes for gene expression and plays an important role in the equilibrium of host development, differentiation, and function. In addition, we can more comprehensively understand the pathogenesis and treatment of IBD by combining different approaches.

### DNA Methylation in IBD

DNA methylation is the most stable and easy to use epigenetic change. It involves the addition of a methyl group at the fifth carbon position of cytosine residue, which is the most common covalent modification. A cut of DNA called CpG island is usually located in a specific region of the genome, such as the promoter or the first exon of a gene. It is rich in CpG dinucleotides but almost always unmethylated. The addition of methyl in DNA methylation is catalyzed by DNA methyltransferase (DNMT), and such as process also depends on dietary substrate and cofactors (Jeltsch et al., 2021). There are many types of DNMTs, such as DNMT1, DNMT3A and DNMT3B. DNMT1 mainly helps maintain the pre-existing DNA methylation profile and methylation pattern, while DNMT3A and DNMT3B are mainly used for *de novo* methylation of unmethylated substrates (Jeltsch et al., 2021).

**FIGURE 2:**
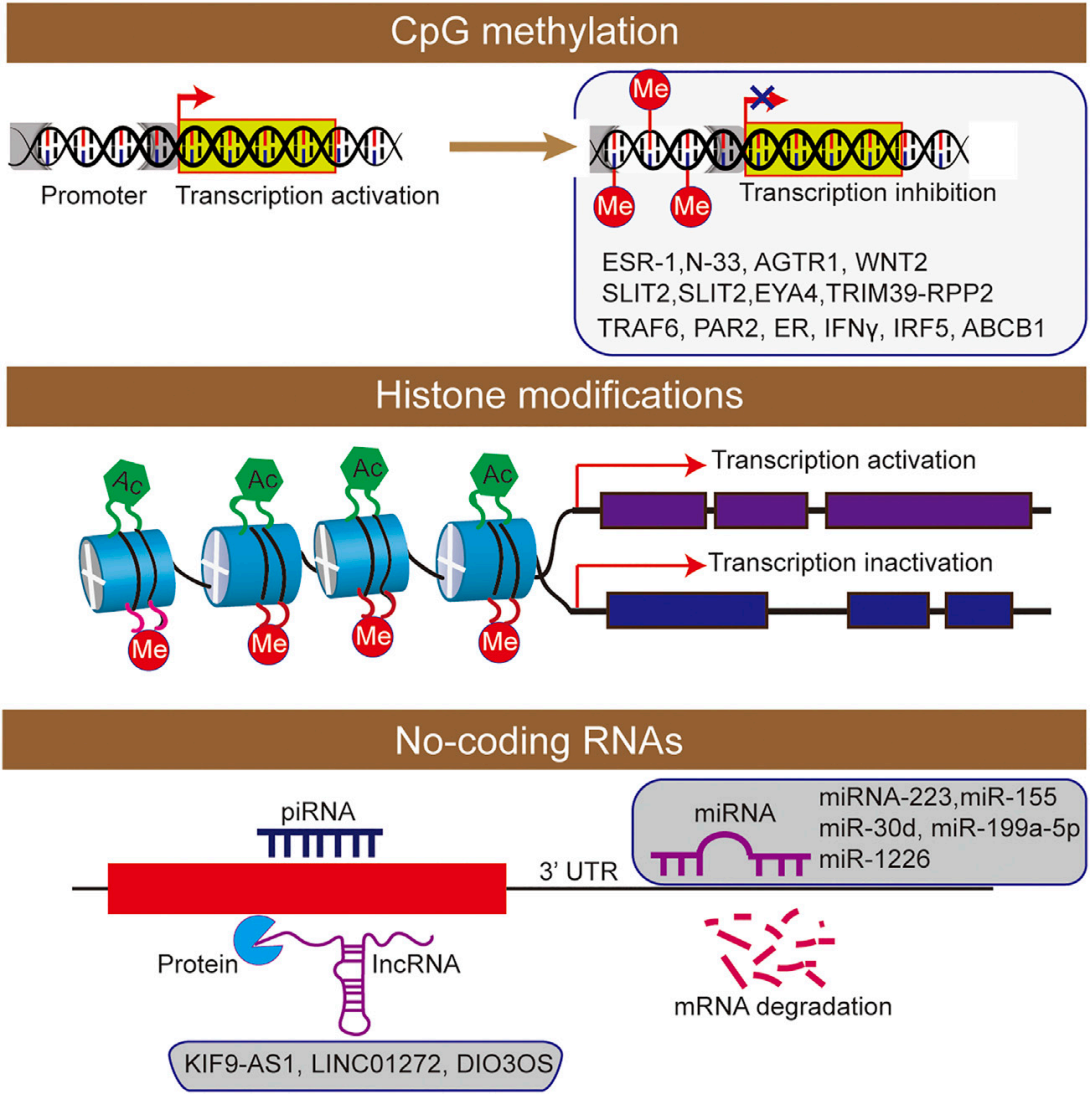
The epigenetic machinery consists of DNA methylation, histone modifications short interfering RNA were participated the regulation of inflammatory bowel disease.

DNA methylation is associated with the addition of methyl groups to nucleotides, and it usually silences gene expression transcriptionally (Quigley, 2012). As the correlation between DNA methylation and IBD pathogenesis have been well established, the epigenetic modification was considered as a key regulatory factor of gene transcription. In fact, DNA methylation in gene promoters was functionally associated with the regulating of gene expression in patients of UC, and providing a new insights into the development and pathogenesis of IBD (Tahara et al., 2009a, Tahara et al., 2009b; Gonsky et al., 2009). With the discovery of 5-hydroxymethylcytosine and its oxidized derivatives in mammalian cells, epigenetic changes such as hydroxymethylation of cytosine have also been confirmed to play an important role in the occurrence and development of diseases (Orr et al., 2012; Chen et al., 2015). However, in inflammatory diseases, knowledge in this direction remains to be explored by scholars. Besides, some DNA methylation analysis methods are mainly based on sodium bisulfite transformation, while they cannot distinguish DNA methylation from DNA hydroxymethylation, and the results may confuse their separate effects (Figure 2).

By analyzing the normal aging colon, researchers have found that the loss of methylated genome gradually occurs with the increase of age (Arasaradnam et al., 2008). This result shows that the methylation status of DNA is related to age. There are also studies showing that age-related methylation accounts for 70% of abnormal gene-specific methylation in the colon. Besides, it is suggested that this is a non-random process, and DNA methylation may be predictable. In addition, the methylation of the tumor suppressor gene ESR1 promoter in the normal human colon is increased with age, which also supports the view that age interferes with the methylation homeostasis of the host (Issa et al., 1994).

It has been mentioned that the DNA methylation process depends on dietary substrates and cofactors, and many studies support that diet may change genome expression and induce host epigenetic modification stably without changing DNA structure (Lee, 2015). For example, the active ingredients in green tea can reverse abnormal methylation. It proves that diet plays an important role in regulating diseases through an epigenetic mechanism. In addition, epigenetic modification is a dynamic process, in which specific dietary factors may cause changes in tissue function, and it is beneficial to the development of diseases. Studies on dietary auxiliary factors and methyl donors have further confirmed the correlation between diet and epigenetic modification (Fernandez-Twinn et al., 2015; Navarro et al., 2017). Besides, vitamin D condition was considered as one of the environmental or dietary associations with the activity of IBD (Lim et al., 2005). And a study found that the monoubiquitination of histone H2B can promote the inflammation through decreased Vitamin D Receptor activity, and which maybe the biomarker of whether to receive Vitamin D supplements of IBD patients (Kosinsky et al., 2021).

The composition of intestinal flora is dynamic and will change with age, environment and the quantity and quality of components in the diet. A large number of studies have shown the close relationship between intestinal flora and disease occurrence and development. For example, a recent study has linked the intake of polyunsaturated fatty acids with differentially methylated CpG related to metabolism and inflammation (Arpón et al., 2017). The diet lacking fiber will lead to the decrease of short-chain fatty acids (SCFAs) from flora, resulting in the change of epigenetic mechanism (Krautkramer et al., 2016). Another study showed the effects mediated by microbiota on maturation of DNA methylation characteristics and transcriptome changes. They found that gut microbiota can dynamically regulate the intestinal transcriptome during postnatal development and targets part of microbially responsive genes by corresponding DNA methylation condition (Pan et al., 2018). Consistently, Camp et al. found microbiota only targets specific genes, instead of changing the all chromatin structure to lead gene expression (Camp et al., 2014). In conclusion, the role of intestinal flora and its metabolites in the epigenetic mechanism of diseases remains to be explored.

Many clinical and basal studies showed that epithelial-mesenchymal transition (EMT) contributes to the pathogenesis and development of IBD (Flier et al., 2010; Scharl et al., 2011). For instance, CDH1, a gene that encoding epithelial cells marker E-cadherin, was downregulated in the area of active UC (Karayiannakis et al., 1998). And another cadherin protein encoding gene CDH13 was methylated in colorectal cancer, which is the one of the complication of UC (Wang et al., 2012). In addition, a study showed that the activity in the methylation condition of EMT-related genes is related to the severity of clinical phenotypes in UC (Tahara et al., 2014). Indeed, they found that several positive associations between hypermethylation of EMT-related genes including CDX1, miR-1247, CDH1, and the severity of clinical UC phenotypes like refractory and severe Mayo endoscopic subscore. These also showed the potential biomarker value of hypermethylation genes, but more intensive study still be needed to perform.

As a unique disease of humans, IBD has a complex pathogenesis. Many factors may be involved in the occurrence and development of IBD. Epigenetics is an important research direction in the pathogenesis of IBD, and its dynamic and reversible characteristics are beneficial to the classification, diagnosis and treatment of IBD. In the epigenetic study of IBD, DNA methylation is one of the most studied directions (Ventham et al., 2016; Howell et al., 2018; Somineni et al., 2019; Gasparetto et al., 2021). The first study on the methylation status of IBD patients was reported in 1996 (Glória et al., 1996). It is found that the DNA level of rectal mucosa in UC patients is lower compared with the normal control group. Compared with inactive UC patients, the DNA methylation level in active UC patients is lower. These results indicate that colon inflammation or mucosal proliferation in IBD patients is related to the hypomethylated DNA spectrum. However, compared with healthy people, UC patients have relative hypermethylation of genome (Arasaradnam et al., 2010). The results of two studies on the methylation status of UC patients are inconsistent, which may be attributed to different methods for quantifying methylation of genomic DNA and different proportion of UC patients in the active stage. The accurate and specific methylation characteristics of IBD patients still need to be studied and determined. The treatment of anti-inflammation like melatonin, probiotics, can regulate the DNA methylation status of intestinal epithelial cells and colitis model (Zhang et al., 2017; Mannino et al., 2019). Recently, the study of newly diagnosed IBD patients in pediatrics shows that the epigenetic characteristics of colon mucosa in UC patients are significant, and most of them disappear after the treatment-induced mucosal inflammation is reduced (Harris et al., 2014). This finding indicates that most DNA methylation changes in untreated pediatric IBD patients are secondary to the inflammatory process of UC. Moreover, abnormal DNA methylation will no longer exist when the disease activity is decreased. Besides the non-specific changes caused by inflammation, cellular heterogeneity may also be one of the reasons for the low specificity of methylation characteristics in IBD patients compared with the control group.

Some studies have focused on the changes of enzymes that catalyze DNA methylation, especially DNMT. Franke et al. have found that IBD is related to the genetic polymorphism of the DNMT3A gene (Franke et al., 2010). In addition, the expressions of DNMT1 and DNMT3B in inflammatory mucosa of UC patients at the active stage are higher compared with samples of the corresponding quiescent stage (Saito et al., 2011). In general, the results indicate that DNA methylation may play an important role in the pathogenesis of IBD.

At present, most studies on DNA methylation focus on the specific methylation characteristics of host genes. In UC patients, the DNA methylation changes of gene promoters are related to the regulation of gene expression. However, the effect of UC on gene-specific methylation in inflammatory mucosa is not clear, and most of them focus on methylation changes of colorectal cancer (CRC)-related genes. Most studies on UC have reported the hypermethylation of ESR-1 and N-33 genes, suggesting their correlation with UC. Besides, some studies have revealed that the DNA methylation levels of three genes AGTR1, WNT2 and SLIT2 in UC patients are increased, which is related to the increased risk of tumor development (Koizumi et al., 2012). Hypermethylation of SLIT2 and EYA4 genes can be used as markers for the early identification of tumors or dysplasia in IBD patients (Azuara et al., 2018). Moreover, some candidate gene studies have described new differentially methylated genes mainly involved in innate immunity in IBD patients, including PAR2, ER, IFNγ, IRF5, ABCB1 (Tahara et al., 2009a; Gonsky et al., 2009; Balasa et al., 2010).

**FIGURE 3:**
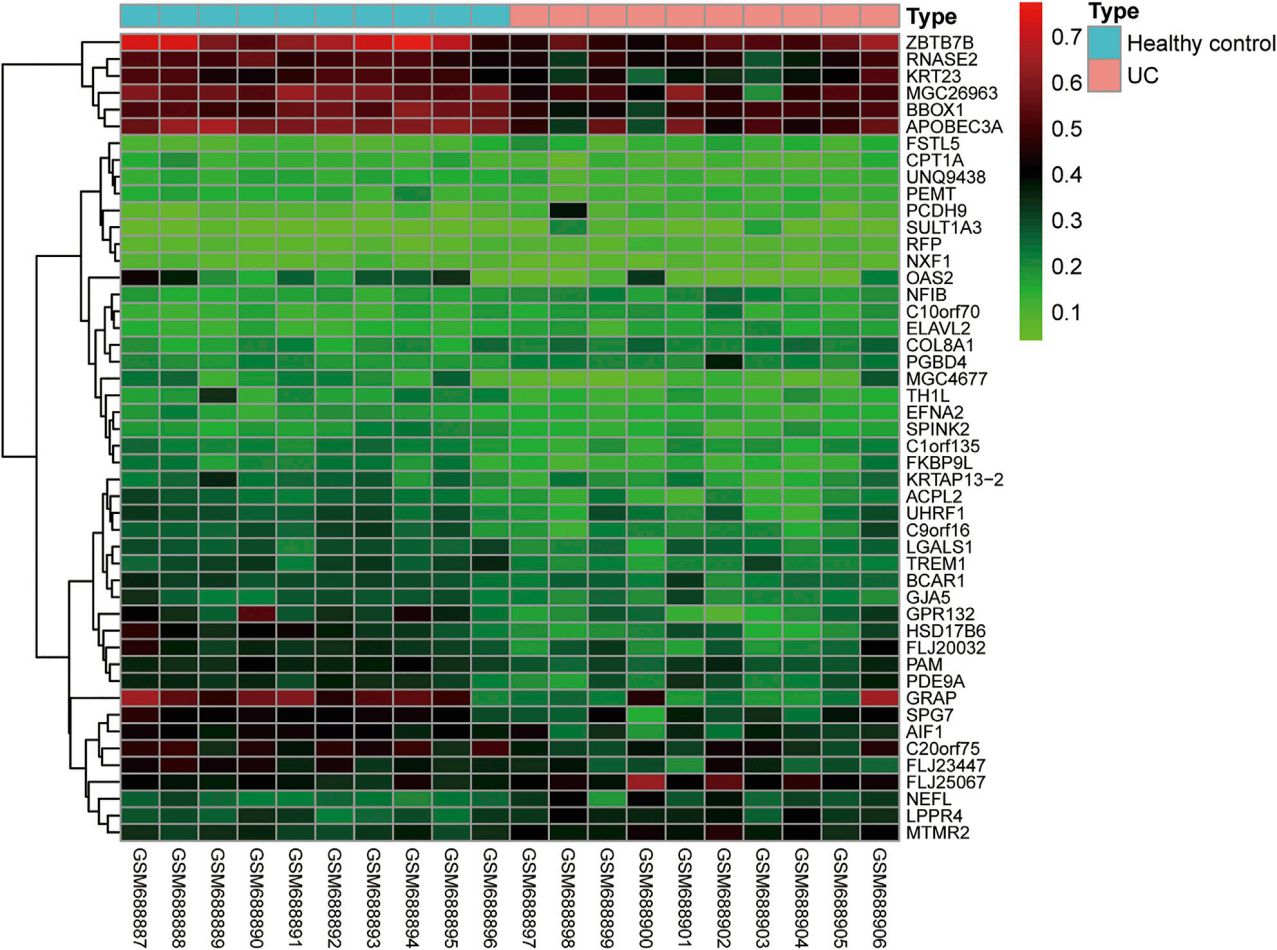
The differently expressed DNA methylation compared UC samples with normal samples.

In addition to identifying the progression of IBD by the methylation status of individual genes, other researchers have indicated that we can conduct total epigenome-wide association studies (EWAS)-related research using genome-wide related research methods to observe the correlation between disease progression and differentially methylated genes in IBD patients. By using a high-throughput DNA methylation method to detect peripheral blood mononuclear cells of IBD patients, a previous study has shown that there is an IBD-related differentially methylated region (DMR) in the TRIM39-RPP2 promoter region, which is proved to be hypomethylated in the colon of pediatric UC patients. Meanwhile, this study has also shown that hypermethylation of TRAF6 in IBD patients is related to the decreased expression of the TRAF6 gene in peripheral mononuclear cells (McDermott et al., 2016). Besides, EWAS can detect the changes in methylation status in peripheral blood and intestinal tissues of CD patients. Researchers have identified many significantly altered methylation sites, and verified that these differentially expressed methylation sites are different in transcription through a posterior cohort (Häsler et al., 2012; Nimmo et al., 2012). In addition, some researchers have shown that the inflammatory and non-inflammatory parts of the rectum in UC and CD patients have different DNA methylation characteristics (Cooke et al., 2012). We found that the DNA methylation profiles of GSE27899 (including 10 UC samples and 10 healthy controls) was obtained from GEO databases, and revealed 48 differential DNA methylation (fold change (FC) > 2 and adj *p* < 0.05) (Figure 3). In general, different disease types and different disease activities will show different DNA methylation characteristics, and a specific methylation characteristic as a non-invasive biomarker will be of great significance to the diagnosis of IBD. However, due to the heterogeneity of analyzed cells, disease subtypes and individuals, the methylation profiles in these studies are difficult to repeat and have little consistency. To solve this problem, it is necessary to refine the grouping and unify the organization sources when analyzing the results, to obtain more realistic methylation characteristics.

### Histone Modification in IBD

Histone modification plays an important role in the occurrence and development of IBD (Rosen et al., 2011; Tsaprouni et al., 2011; Scarpa and Stylianou, 2012). The nucleosome is the basic structural unit of chromatin, and histone and 147 base pairs of the DNA combine to form nucleosome structure (Johnstone, 2002). Histones mainly include five types: H2A, H2B, H3, H4 and H1. As the main protein component of chromatin, histones play a role in gene regulation. When histone is loosely attached to DNA, the chromatin formed at this time is called euchromatin, and transcription factors can contact DNA very well. When histone is compressed, it forms heterochromatin with DNA. Moreover, the chromatin at this time is equivalent to silent chromatin, preventing transcription factors from approaching DNA. There are various types of histone modification, and 12 types have been identified at present (Johnstone, 2002), including acetylation, methylation, ubiquitination, phosphorylation, glycosylation, or citrullination (Figure 2).

Among the post-translational histone modifications, acetylation and methylation are the most studied post-translational modifications (Zhang et al., 2015). The acetylation and deacetylation of histone are mainly catalyzed by two enzymes, namely histone acetyl transferases (HATs) and histone deacetylases (HDACs). These two enzymes play an important role in cell function, especially cell proliferation and apoptosis. In addition, there is a positive correlation between histone modification and gene expression, and the research mainly focuses on lysine acetylation at the tail of H3 and H4 (Gardner et al., 2011). When histone is acetylated, chromatin is loose, which is related to transcription activity. When histone is deacetylated, chromatin is compressed, and the transcription activity is decreased, suggesting that the acetylation state of histone can be regulated by modulating the above two-mentioned enzymes to control the function of the host. There is little research on post-translational histone modification in IBD. In the DSS-induced colitis model, HDAC inhibitor can increase the expression of the Foxp3 gene and improve the inhibitory function of regulatory T cells in mice with colitis, thus alleviating the inflammation phenotype (de Zoeten et al., 2010). In the mouse model of colitis induced by another chemical agent, HDAC inhibitor can induce apoptosis and inhibit pro-inflammatory factors, thus playing a protective role. It is also found that in the inflammatory mucosa of mice with colitis, histone 4 acetylation is higher compared with non-inflammatory tissues, while the causal relationship between H4 acetylation and inflammatory activation remains largely unclear. However, the data related to IBD are controversial, and a lot of research is needed to further clarify it. The balance between HDACs and HATs may be related to IBD, whereas the adjustment of this balance is not clear.

In addition, histone modification in IBD may be an indicator of the interaction between intestinal microorganisms and the host. For example, the SCFAs produced by most symbiotic bacteria, such as *Akkermansia muciniphila*, *Clostridium butyricum*, and *Faecalibacterium prausnitzi*, in the intestinal tract of the host are considered as an HDAC inhibitor, which can inhibit HDAC activity by increasing HAT activity, while they possess anti-inflammatory effect and epithelial barrier maintenance effect in various animal models (Lukovac et al., 2014). Moreover, they can inhibit the differentiation of pro-inflammatory macrophages in a manner related to HDAC (Lukovac et al., 2014).

In histone methylation, methyl groups are added to lysine and spermatic acid by histone methyltransferase, thus inhibiting or activating transcription activity according to the position of targeted amino acids. The targeted enzyme may regulate abnormal gene expression and play a regulatory role in the occurrence of diseases. However, at present, it is unclear whether histone modification is the primary or secondary change. The correlation between the abnormal composition of intestinal flora and the occurrence and development of IBD has been confirmed by a large number of studies. Recent studies have found that there is a correlation between the microbial composition of colon mucosa and DMR in IBD patients (Kraiczy et al., 2016). For example, the decreased abundance of *Roseburia* in the intestinal tract of UC patients is related to the reduced methylation of the KHDC3L gene. These results suggest that epigenetics and intestinal flora may work together to regulate the occurrence of IBD.

### NcRNA in IBD

NcRNA is an important discovery in epigenetics, which can be divided into long ncRNA, medium ncRNA, short ncRNA, and microRNA (miRNA) according to the length (Nie et al., 2012). NcRNA is involved in cell proliferation and differentiation, which can interfere with mRNA translation and induce mRNA degradation, or regulate gene expression through interaction with DNA or protein (Aalto and Pasquinelli, 2012; Nie et al., 2012). In addition, the importance of miRNA in IBD also be interpreted by studies (He et al., 2016; James et al., 2020). In conclusion, ncRNA research plays an essential role in the mechanism of IBD.

#### lncRNA in IBD

The expression profile can successfully distinguish IBD patients from healthy controls. The transcription characteristics and clinically relevant parameters of lncRNA indicate that lncRNA has the potential as a biomarker of IBD (Mirza et al., 2015). Another study also found differences expression levels of various lncRNAs (KIF9-AS1, LINC01272 and DIO3OS) were detected in tissue and plasma samples of patients with IBD (Wang et al., 2018). Subsequently, receiver operating characteristic (ROC) curve analysis was used to determine the specificity and sensitivity of these lncRNAs, and identify it as potential diagnostic biomarkers for IBD.

#### The Correlation Between miRNA and IBD

As one of the most studied mechanisms in IBD, miRNA is an endogenous single-stranded ncRNA consisting of 17–25 nucleotides, which is folded by single-stranded RNA to form a short hairpin structure. It is detected in introns, exons, or regulatory sequences of genomes, which are mainly located in intergenic regions (Lee et al., 1993; Galatenko et al., 2018). The miRNA recognizes mRNA target sites (usually in 3′ untranslated regions) through complementary base pairing, thus regulating inflammatory response and release of inflammatory factors (Kim et al., 2017; Gruszka and Zakrzewska, 2018; Perconti et al., 2019). Gene can be regulated by miRNAs, some of which can regulate DNA methylation or histone modification. For instance, Fabbri et al. show a regulatory function of miR-29s on DNMT3A, which led to the inhibition of tumorigenicity (Fabbri et al., 2007). On the contrary, these two epigenetic processes can also regulate the production of miRNAs.

**FIGURE 4:**
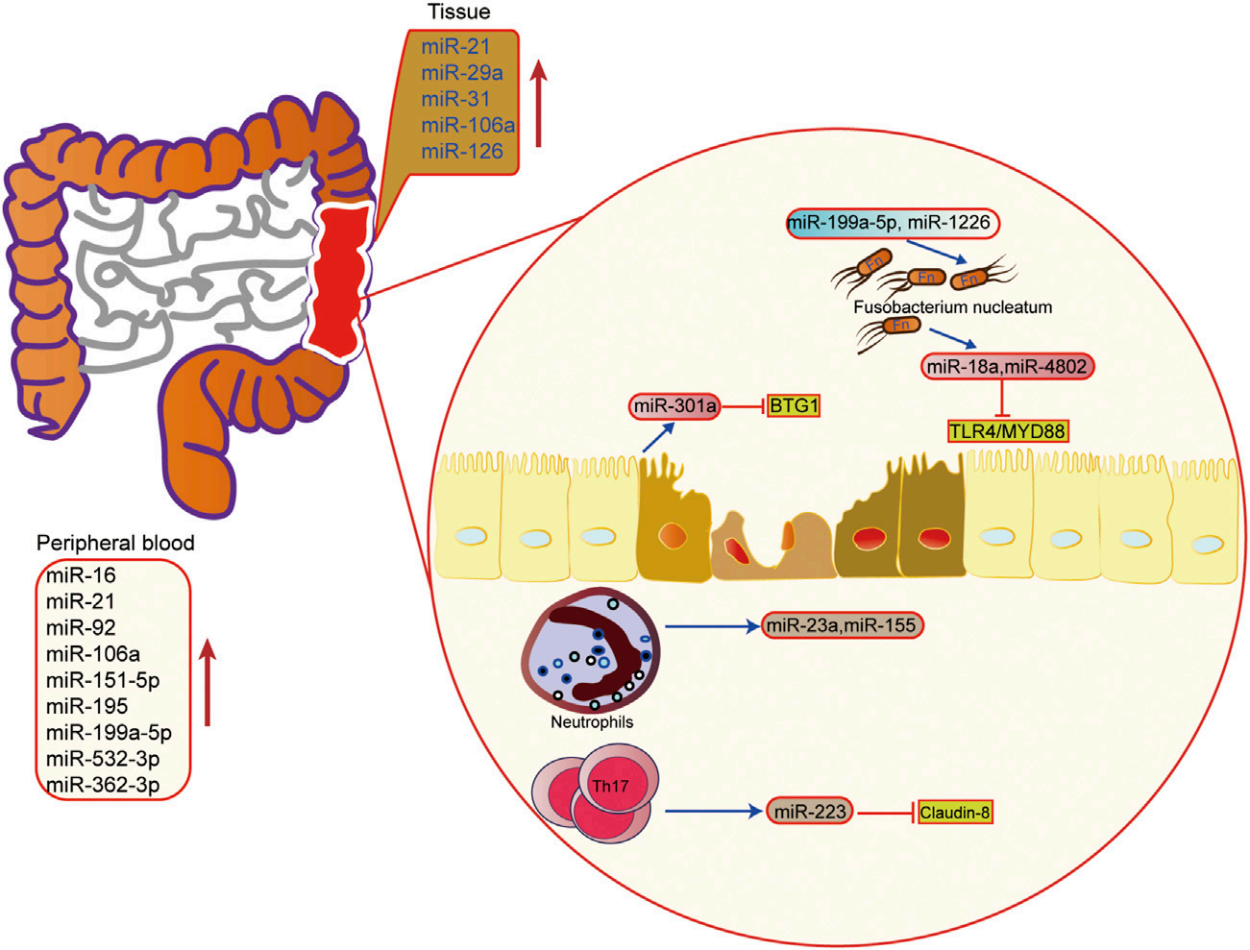
The role of microRNAs in epigenetic regulation in inflammatory bowel disease. The upregulated microRNA in inflammatory bowel disease tissue and peripheral blood. miRNA also mediated the interaction of microbiota microbiome, immune cells and host cells in IBD.

In IBD, miRNA is involved in regulating the pathogenesis of UC, including regulation of immune cells, intestinal epithelial barrier, and the homeostasis between the intestinal flora and host. It has been found that miRNA is the key regulator of intestinal immunity, and it participates in innate immunity and adaptive immunity. In response to inflammation, miRNA can influence the maturation and differentiation of immune cells. For example, the miRNA-223 derived from bone marrow can reduce the release of IL-1β by inhibiting NLRP3, thus alleviating mouse colitis (Neudecker et al., 2017). When intestinal macrophages and dendritic cells lack miR-223, a pro-inflammatory phenotype will appear (Zhou et al., 2015). In addition, when the exosomes containing miRNA-155 are released into the intestinal tract, the host macrophages are induced to polarize towards M1, and colitis is aggravated (Wei et al., 2020). Other studies have found that activated neutrophils can promote the release of miR-23a and miR-155, which play a pro-inflammatory role (Figure 4) (Butin-Israeli et al., 2019).

The intestinal barrier and intestinal flora are also important links in the pathogenesis of IBD. It is found that miRNA can also regulate the intestinal epithelial barrier and participate in the steady-state regulation between intestinal microorganisms and the host. Activation of the Th17 pathway can induce the release of miR-223 and then target Claudin-8 to destroy the tight junction in the intestinal epithelial barrier (Wang et al., 2016a). In the aspect of intestinal flora homeostasis regulation, researchers have found that *Fusobacterium nucleatum* can inhibit the expressions of miR-18a and miR-4802 by activating TLR4/MYD88 pathway, and then reduce the inhibition of miRNA on its target gene to activate the process of autophagy (Yu et al., 2017). SCFAs, one of the metabolites of intestinal microbes, can also promote the expression of miRNA in B cells, and regulate the differentiation of B cells (Sanchez et al., 2020). In addition, the host can also regulate the structure and growth of intestinal flora through miRNA. Liu et al. have shown that miR-30 d in feces can target *Akkermansia muciniphila* and increase the abundance of modified bacteria by up-regulating lactase expression (Liu et al., 2019). Besides, some miRNAs, such as miR-199a-5p and miR-1226, can regulate the proliferation of *Fusobacterium nucleatum* and *segmental filamentous bacteria* (Ji et al., 2018). These findings show that miRNA plays an important role in the interaction between host and intestinal flora, providing new ideas for maintaining intestinal homeostasis.

#### The Role of miRNA in the Diagnosis, Monitoring and Prognosis of IBD

At present, colonoscopy and pathological examination are the gold standards in the diagnosis of UC, while there are some limitations in the early diagnosis of UC due to their invasiveness and the possibility of complications. Therefore, we need to find a non-invasive and more convenient method to diagnose and monitor the prognosis of UC. By analyzing the miRNAs in feces, blood, and tissue samples, the researchers have indicated that some miRNAs can be used as biomarkers (Figure 3) (Wu et al., 2011; Zahm et al., 2011; Oikonomopoulos et al., 2016). For example, the expressions of miR-21 and miR-92a in UC plasma are up-regulated, which can well distinguish active UC from healthy controls and IBD patients. The specificity of the two indicators is 92 and 100%, respectively, and the sensitivity is 88% (Oikonomopoulos et al., 2016). However, the expression of miR-21 is positively correlated with the severity of the disease and closely related to the development of inflammation. In addition, miR-375 is also reported to be highly expressed in the plasma of UC patients. Contrary to its low expression in colon tissues of UC patients, this well supports the role of miR-375 as a potential marker for UC diagnosis. Basic research has also found that miR-375 can affect the growth and invasion of colon cells, and overexpression of miR-375 will enhance apoptosis and necrosis (Alam et al., 2017; Garrido-Mesa et al., 2018). Moreover, miR-19b is found to be down-regulated in active CD. Among them, cytokine signaling inhibitor 3 (SOCS3) is predicted as a potential target. Further examination using intestinal tissue samples has verified that miR-19b is negatively correlated with the expression of SOCS3 at the protein level in active CD patients (Cutler et al., 2015). Besides, the expression of miR-10a in IBD inflammatory mucosa is significantly decreased, showing that it plays a role by targeting NOD2 and IL-12/IL-23p40 (Levine et al., 2013).

One of the main features of IBD is that it is prone to recurrent attacks, which is of great significance for monitoring IBD during its active stage and evaluating its prognosis. Some studies have shown that miRNA in plasma and feces is closely related to the activity of IBD. For example, miRNA-146a has been reported to be down-regulated in UC, which is significantly correlated with disease activity index and endoscopic activity. According to basic research, the miRNA-146a may alleviate colon inflammation by targeting TRAF6 and NF-κB signaling pathways (Wang et al., 2019; Garo et al., 2021), and it can inhibit the activation of pro-inflammatory M1 macrophages, as well as the production and release of pro-inflammatory factors through the TLR4 pathway (Deng et al., 2019). Moreover, the results show that the expression of miR-223 is positively correlated with the disease activity of UC. MiR-223 in feces can well distinguish IBD patients in the active stage and remission stage, and the sensitivity and specificity are 80 and 93%, respectively (Schönauen et al., 2018). Besides, some studies have evaluated the response of IBD patients to treatment according to the expression of miRNA. By screening the responses to anti-TNF-α and glucocorticoid treatment, researchers have selected five miRNAs related to the treatment response from serum (Batra et al., 2020). A prospective study by Kalla et al. has reported that miR-3615 and miR-4792 in blood T cells contribute to the prognosis of UC (Kalla et al., 2020). However, most of the current studies only focus on detecting the changing trend of miRNA expression, and more quantitative analyses still need to be carried out.

### The Epigenetic Mechanism of T Cells, B Cells and IECs in IBD

#### T Cells

Helper T (Th) cells have been identified as key participants in IBD. Cytokines are necessary participants for helper T cells to differentiate into subtypes, and epigenetic modification plays an important role in such a process. Studies have shown that DNA methylation regulates cytokine expression during helper T cell differentiation, thus regulating signal transduction and tissue factor network, and also promoting the maturation of cytokines in Th1 cells, Th2 cells, and Th17 cells (Stadhouders et al., 2018). Meanwhile, a large number of studies have explored the changes of DNA methylation and histone modification level of the *IFNG* locus during Th1 differentiation and IL4 locus during Th2 differentiation (Nielsen and Tost, 2013). Gonsky et al. found that IBD patients who required surgery had decreased IFNG methylation, compared with that of non-surgical patients. And the decreasing IFNG methylation level was negatively correlated with the level of IFN-γ in UC patients (Gonsky et al., 2011). Histone modification can impact the acting effector cytokines of Th17 cells (Hagihara et al., 2019). And TGFβ and IL6 can regulate the transfer of Th1 cells to Th17 cells by regulating Runx1 expression and H3K9 acetylation (Ueno et al., 2015). Another study found that the deficiency of one of the histone acethyltransferases can downregulate Th17-associated genes, even in a DSS-induced colitis model (Yang et al., 2018). In addition, regulating the expression of the transcription factor Foxp3 by changing the chromatin histone acetylation condition may be the key of Treg function, which maybe have an important effect on regulating IBD (von Knethen et al., 2020). Consistently, three histone modification of the Foxp3 protein can affect the stability, and then regulate Treg cells function and the development of IBD (Dominguez-Villar and Hafler, 2018).

LncRNA and miRNA also plays an important role in the differentiation and development of Th cells. *Nest* is a lncRNA gene which expressed in immune cells and seemed to be involved in inflammation. Studies showed Nest can alter the expression of IFN-γ, and the expression of Nest was associated with severity of UC patients (Gomez et al., 2013; Padua et al., 2016). In addition, miRNA can also regulate the differentiation and function of Tregs, Th17 cells, Th1 cells and other immune cells in adaptive immunity. Studies showed that miRNAs can influence the development of Th17 cells or differentiation into regulatory T cells by targeting some genes, such as BCL6, STAT1 and SOCS1 (Urich et al., 2015). Besides, some miRNAs like miR-22 can promote the differentiation of Th17 cells and it had a increasing level in the peripheral blood and intestinal mucus of IBD patients (Pei et al., 2018). And miR-155 can induce Th17 differentiation by targeting Jarid2 (Xu et al., 2017), the colitis model in miR-155-deficient mice had a decreasing levels of Th1 cells, Th17 cells, dentritic cells, which compared with WT mice (Singh et al., 2014). Macrophages will differentiate into M2 phenotype when they lack miR-155, and inhibit the differentiation of Th1 and Th17 cells (Hou et al., 2017; Li et al., 2018); and miR-155, miR-34a, miR-18a, miR-7 can also contribute to stabilize the suppressor function of Tregs (Fayyad-Kazan et al., 2012; Dooley et al., 2013; Lu et al., 2015). In addition, miR-21 can also promote the differentiation and function of Th2 cells (Murugaiyan et al., 2015; Wang et al., 2016b). Furthermore, miR-21 can regulate the expression of Foxp3, STAT3, STAT5, which involved in the imbalance of Th17 cells and Tregs (Dong et al., 2014).

### B Cells

B cells in intestinal mucosa can produce immunoglobulin, providing the first line of defense for host immunity (Brandtzaeg, 2010). Studies on the correlation between IBD and B cells have been reported, and IBD lesions also promote IgG production (Brandtzaeg et al., 2006). It has been found that the level of B cells in the blood of IBD patients is increased, and the expression of TLR2 and IL8 are increased (Noronha et al., 2009). In addition, SCFAs derived from intestinal flora, such as butyric acid, can regulate the transformation of immunoglobulin types by regulating histones and exert immune regulatory effects on hosts (White et al., 2014). The development and differentiation of B cells are also affected by an epigenetic mechanism. For example, the development and differentiation of CD8^+^B cells in adaptive immunity can be regulated by some miRNAs (Xu and Zhang, 2016; Yang et al., 2016). Generally speaking, only very few studies have been carried out on the effect of epigenetics on B cells in IBD. More basic experiments are needed to clarify the influence of epigenetics on B cells to provide a new direction for the research of pathogenesis and treatment of IBD.

Generally speaking, regulating epigenetics to modulate the immune microenvironment may be an effective method to treat IBD, while its effectiveness and accuracy still need a lot of experiments. Moreover, the causal relationship between epigenetics and immune cell differentiation or functional changes in IBD patients is still unclear. There are many targets in epigenetic mechanisms, especially miRNA. The anti-inflammatory effect may be the comprehensive expression after acting on a variety of immune cells. Therefore, the specific regulatory mechanism of epigenetics needs to be explored.

### Intestinal Epithelial Cells

The single layer of intestinal epithelial cells and the tight junctions between them form the mucosal barrier. The intestinal epithelial cells are well-organized and each performs a different function, including producing mucin to defend luminal microbes, facilitating nutrient absorption. A study reported the result of epigenomics sequencing from matched inflamed and non-inflamed mucosa of colon, and found that classification of disease can be improved by epithelial DNA methylation. And the characteristics of epithelial DNA methylation also was associated with inflammation and gut microbiota (Ryan et al., 2020). In addition, the epigenetic regulation disorder happened in the intestinal epithelial cells is also important in the pathogenesis and development of IBD. Liu et al. found that SETD2, which is a trimethyltransferase of histone H3K36, can regulate oxidative stress to relieve inflammation in mice (Liu et al., 2021). Specifically, increasing susceptibility to DSS-induced colitis would be came out after specific knockout Setd2 in villus cells. Besides, the expression of miR-301a is up-regulated in the intestinal epithelium of active IBD patients. Studies also show that miR-301a can reduce the expression of cadherin-1 by targeting BTG1, thereby destroying the intestinal barrier function and promoting the occurrence of inflammation and tumor (He et al., 2017).

## Multi-Omics Approaches Identifies Epigenetic Modification in IBD

Epigenetics is heritable and reversible, and it participates in the development, differentiation balance and function of host cells (Wawrzyniak and Scharl, 2018). Epigenetics also plays an important regulatory role in the occurrence and development of IBD influenced by many factors, which help improve the cognition of disease pathogenesis and provide new ideas for diagnosis and treatment. However, it is still not enough to explain the pathogenesis of IBD by a single factor.

At present genome-wide techniques, such whole-genome bisulfite-seq were used for DNA methylation profiles. As chromatin modification and accessibility are another important aspect of epigenetic changes. One of the most widely used techniques to capture the accessibility of chromatin is called the use of sequencing-accessible chromatin analysis (ATAC-seq), DNase-seq and FAIRE-seq, which can be used to detect chromatin binding Sites and specific transcription factors. Other techniques, such as array-based and sequencing-based methods, ChIP-chip and ChIP-seq are used to identify post-translational modifications of histones bound to DNA regions or domains, including methylation, acetylation, etc.

Multi-omics integration analysis has emerged with the wide application of high-throughput technologies. Researchers can obtain large-scale omics data from different molecular levels such as genome, transcriptome, proteome, interactionome, epigenome, single-cell multiomics and microbiome. Multi-omics integrated data analysis has revolutionized biology and promoted our deep understanding of the biological processes and molecular mechanisms of IBD. Meanwhile, the unbiased data-driven integration strategy and the powerful bioinformatics tool will be favor to integrate multiple-omes (de Souza et al., 2017). For example, Satangi *et al.* have identified the DMRs in patients with primary CD from peripheral blood leukocytes. These DMRs and IBD-susceptible single nucleotide polymorphisms (SNPs) are significantly co-located, indicating that their genetic origins may be consistent (Adams et al., 2014). And the results show the interaction between genome and epigenetic group, suggesting that genetic changes promote epigenetic changes (Richards, 2006). In addition, Hasler and his colleagues have identified 61 disease-related genes in 20 pairs of monozygotic twins by combining transcriptomics, differential methylation region assessment and genome-wide methylation quantitative variable position assessment (Häsler et al., 2012). They have shown that most genes play a role in the immune process, which is consistent with previous studies on the genetic mechanism of UC. The results demonstrate the ability to explore the pathogenesis of UC by combining transcriptome and methylation providing a novel idea for the study of UC. In addition, the analysis for epigenome regulation at the single-cell level is the forefront of the omics in the epigenetic field. For instance, the single-cell multiomics can profile transcriptome and histone modification or open chromatin simultaneously, to reveal the dynamic changes in gene regulation (Harada et al., 2021). Generally speaking, the interaction of multiple groups may be more conducive to exploring the pathogenesis of IBD providing a new choice for disease prevention, early diagnosis and treatment.

## Conclusion

More and more research support the importance of non-genetic factors, especially epigenetic factors, in the pathogenesis of IBD. DNA methylation is one of the most stable and abundant mechanisms in epigenetics research, which is of great significance to study the pathogenesis of IBD. However, DNA methylation may be affected by other factors, thus resulting in non-specific changes. Setting control will help improve this problem. Moreover, refined sample processing can make the methylation changes of different cell types clearer. At present, it is not clear whether histone modification is a primary or secondary epigenetic change. A combination of histone modification and DNA methylation may be more beneficial for exploring the pathogenesis of IBD. Intestinal flora is a key component of the intestinal environment. In the future, it will be a new direction to study the interaction between mucosal flora and an epigenetic group of host epithelium by co-culturing organoids or intestinal stem cells with specific microorganisms. It also provides theoretical support for specific microorganism-oriented prevention and treatment interventions.

The research on diagnosis and treatment based on epigenetics is increasing. Targeting the exposed sites of the epigenetic transmission environment and using specific epigenetic changes of IBD for diagnosis and treatment will be beneficial to the prevention, diagnosis, and treatment of IBD. Further clinical transformation still needs large-scale clinical trials to prove. In general, the correlation with IBD emphasizes the significance of epigenetics in IBD, and the discovery of related work will provide new ideas for targeted epigenetics, as well as prevention and treatment measures of intestinal flora.

## Glossary

AGTR1: angiotensin II receptor type 1
ABCB1: ATP binding cassette subfamily B member 1
BTG1: BTG anti-proliferation factor 1.
CD: Crohn’s disease
CDH: cadherin
CDX1: caudal type homeobox-1
CRC: colorectal cancer
DNMT: DNA methyltransferase
DMR: differentially methylated region
DSS: dextran sodium sulfate
EWAS: epigenome-wide association studies
ESR: erythrocyte sedimentation rate
EMT: epithelial-mesenchymal transition
ER: estrogen receptor
EYA4: EYA transcriptional coactivator and phosphatase 4
FC: fold change
Foxp3: forkhead box P3
GEO: Gene Expression Omnibus
HATs: histone acetyl transferases
H3K9: histone 3 lysine 9
H3K36: histone 3 lysine36
HDACs: histone deacetylases
IFNγ: interferon gamma
IRF5: interferon regulatory factor 5
IBD: Inflammatory bowel disease
lncRNA: long noncoding RNA
IL-8: interleukin 8
IL4: interleukin 4
IL6: interleukin 6
IL-1β: interleukin 1 beta
IL-12: interleukin 12
IL-23: interleukin 23
miRNA: microRNA
MYD88: myeloid differentiation primary response gene 88
NLRP3: NLR family pyrin domain containing 3
NF-κB: nuclear factor kappa B
ncRNA: non-coding RNA
NOD2: nucleotide binding oligomerization domain containing 2
PAR2: F2R like trypsin receptor 1
Runx1: RUNX family transcription factor 1
SLIT2: slit guidance ligand 2
SCFAs: short-chain fatty acids
SNPs: single nucleotide polymorphisms
STAT: signal transducer and activator of transcription
SETD2: SET domain containing 2
SOCS3: cytokine signaling inhibitor
TRAF6: TNF receptor associated factor 6
Th17: T helper 17: T helper 17
TLR4: toll like receptor 4
TNF-α: tumour necrosis factor alpha
Th1: T helper 1
Th2: T helper 2
Th17: T helper 17: T helper 17
TGFβ: transforming growth factor beta
Tregs: regulatory T cells
TLR2: toll like receptor 2
UC: ulcerative colitis
WNT2: Wnt family member 2
WT: wild type.

## References

[B1] Aalto A. P. Pasquinelli A. E. Small Non-coding RNAs Mount a Silent Revolution in Gene Expression. Curr. Opin. Cell Biol 2012;24:333–340. 10.1016/j.ceb.2012.03.006 22464106 PMC3372702

[B2] Adams A. T. Kennedy N. A. Hansen R. Ventham N. T. OʼLeary K. R. Drummond H. E. Two-stage Genome-wide Methylation Profiling in Childhood-Onset Crohn's Disease Implicates Epigenetic Alterations at the VMP1/MIR21 and HLA Loci. Inflamm. Bowel Dis. 2014;20:1784–1793. 10.1097/MIB.0000000000000179 25144570 PMC4736293

[B3] Alam K. J. Mo J. S. Han S. H. Park W. C. Kim H. S. Yun K. J. MicroRNA 375 Regulates Proliferation and Migration of colon Cancer Cells by Suppressing the CTGF-EGFR Signaling Pathway. Int. J. Cancer 2017;141:1614–1629. 10.1002/ijc.30861 28670764

[B4] Arasaradnam R. P. Commane D. M. Bradburn D. Mathers J. C. A Review of Dietary Factors and its Influence on DNA Methylation in Colorectal Carcinogenesis. Epigenetics 2008;3:193–198. 10.4161/epi.3.4.6508 18682688

[B5] Arasaradnam R. P. Khoo K. Bradburn M. Mathers J. C. Kelly S. B. DNA Methylation of ESR-1 and N-33 in Colorectal Mucosa of Patients with Ulcerative Colitis (UC). Epigenetics 2010;5:422–426. 10.4161/epi.5.5.11959 20505342

[B6] Arpón A. Milagro F. I. Razquin C. Corella D. Estruch R. Fitó M. Impact of Consuming Extra-Virgin Olive Oil or Nuts within a Mediterranean Diet on DNA Methylation in Peripheral White Blood Cells within the PREDIMED-Navarra Randomized Controlled Trial: A Role for Dietary Lipids. Nutrients, 10 2017;10. 10.3390/nu10010015 PMC5793243 29295516

[B7] Azuara D. Aussó S. Rodriguez-Moranta F. Guardiola J. Sanjuan X. Lobaton T. New Methylation Biomarker Panel for Early Diagnosis of Dysplasia or Cancer in High-Risk Inflammatory Bowel Disease Patients. Inflamm. Bowel Dis. 2018;24:2555–2564. 10.1093/ibd/izy255 30099509

[B8] Balasa A. Gathungu G. Kisfali P. Smith E. O. Cho J. H. Melegh B. Assessment of DNA Methylation at the Interferon Regulatory Factor 5 (IRF5) Promoter Region in Inflammatory Bowel Diseases. Int. J. Colorectal Dis. 2010;25:553–556. 10.1007/s00384-010-0874-0 20127100 PMC5751749

[B9] Batra S. K. Heier C. R. Diaz-Calderon L. Tully C. B. Fiorillo A. A. van den Anker J. Serum miRNAs Are Pharmacodynamic Biomarkers Associated with Therapeutic Response in Pediatric Inflammatory Bowel Disease. Inflamm. Bowel Dis. 2020;26:1597–1606. 10.1093/ibd/izaa209 32793975 PMC7500519

[B10] Brandtzaeg P. Carlsen H. S. Halstensen T. S. The B-Cell System in Inflammatory Bowel Disease. Adv. Exp. Med. Biol. 2006;579:149–167. 10.1007/0-387-33778-4_10 16620017

[B11] Brandtzaeg P. Update on Mucosal Immunoglobulin A in Gastrointestinal Disease. Curr. Opin. Gastroenterol. 2010;26:554–563. 10.1097/MOG.0b013e32833dccf8 20693891

[B12] Burisch J. Munkholm P. The Epidemiology of Inflammatory Bowel Disease. Scand. J. Gastroenterol. 2015;50:942–951. 10.3109/00365521.2015.1014407 25687629

[B13] Butin-Israeli V. Bui T. M. Wiesolek H. L. Mascarenhas L. Lee J. J. Mehl L. C. Neutrophil-induced Genomic Instability Impedes Resolution of Inflammation and Wound Healing. J. Clin. Invest 2019;129:712–726. 10.1172/JCI122085 30640176 PMC6355304

[B14] Camp J. G. Frank C. L. Lickwar C. R. Guturu H. Rube T. Wenger A. M. Microbiota Modulate Transcription in the Intestinal Epithelium without Remodeling the Accessible Chromatin Landscape. Genome Res. 2014;24:1504–1516. 10.1101/gr.165845.113 24963153 PMC4158762

[B15] Chen B. Sun L. Zhang X. Integration of Microbiome and Epigenome to Decipher the Pathogenesis of Autoimmune Diseases. J. Autoimmun. 2017;83:31–42. 10.1016/j.jaut.2017.03.009 28342734

[B16] Chen L. Chen K. Lavery L. A. Baker S. A. Shaw C. A. Li W. MeCP2 Binds to Non-CG Methylated DNA as Neurons Mature, Influencing Transcription and the Timing of Onset for Rett Syndrome. Proc. Natl. Acad. Sci. U S A. 2015;112:5509–5514. 10.1073/pnas.1505909112 25870282 PMC4418849

[B17] Cooke J. Zhang H. Greger L. Silva A. L. Massey D. Dawson C. Mucosal Genome-wide Methylation Changes in Inflammatory Bowel Disease. Inflamm. Bowel Dis. 2012;18:2128–2137. 10.1002/ibd.22942 22419656

[B18] Cutler D. J. Zwick M. E. Okou D. T. Prahalad S. Walters T. Guthery S. L. Dissecting Allele Architecture of Early Onset IBD Using High-Density Genotyping. PLoS One 2015;10, e0128074. 10.1371/journal.pone.0128074 26098103 PMC4476779

[B19] de Souza H. S. P. Fiocchi C. Iliopoulos D. The IBD Interactome: an Integrated View of Aetiology, Pathogenesis and Therapy. Nat. Rev. Gastroenterol. Hepatol. 2017;14:739–749. 10.1038/nrgastro.2017.110 28831186

[B20] de Zoeten E. F. Wang L. Sai H. Dillmann W. H. Hancock W. W. Inhibition of HDAC9 Increases T Regulatory Cell Function and Prevents Colitis in Mice. Gastroenterology 2010;138:583–594. 10.1053/j.gastro.2009.10.037 19879272 PMC3369426

[B21] Deng F. He S. Cui S. Shi Y. Tan Y. Li Z. A Molecular Targeted Immunotherapeutic Strategy for Ulcerative Colitis via Dual-Targeting Nanoparticles Delivering miR-146b to Intestinal Macrophages. J. Crohns Colitis 2019;13:482–494. 10.1093/ecco-jcc/jjy181 30445446

[B22] Dominguez-Villar M. Hafler D. A. Regulatory T Cells in Autoimmune Disease. Nat. Immunol. 2018;19:665–673. 10.1038/s41590-018-0120-4 29925983 PMC7882196

[B23] Dong L. Wang X. Tan J. Li H. Qian W. Chen J. Decreased Expression of microRNA-21 Correlates with the Imbalance of Th17 and Treg Cells in Patients with Rheumatoid Arthritis. J. Cell Mol Med 2014;18:2213–2224. 10.1111/jcmm.12353 25164131 PMC4224555

[B24] Dooley J. Linterman M. A. Liston A. MicroRNA Regulation of T-Cell Development. Immunol. Rev. 2013;253:53–64. 10.1111/imr.12049 23550638

[B25] Fabbri M. Garzon R. Cimmino A. Liu Z. Zanesi N. Callegari E. MicroRNA-29 Family Reverts Aberrant Methylation in Lung Cancer by Targeting DNA Methyltransferases 3A and 3B. Proc. Natl. Acad. Sci. U S A. 2007;104:15805–15810. 10.1073/pnas.0707628104 17890317 PMC2000384

[B26] Fayyad-Kazan H. Rouas R. Fayyad-Kazan M. Badran R. El Zein N. Lewalle P. MicroRNA Profile of Circulating CD4-Positive Regulatory T Cells in Human Adults and Impact of Differentially Expressed microRNAs on Expression of Two Genes Essential to Their Function. J. Biol. Chem. 2012;287:9910–9922. 10.1074/jbc.M111.337154 22294691 PMC3323050

[B27] Fernandez-Twinn D. S. Constância M. Ozanne S. E. Intergenerational Epigenetic Inheritance in Models of Developmental Programming of Adult Disease. Semin. Cell Dev Biol 2015;43:85–95. 10.1016/j.semcdb.2015.06.006 26135290 PMC5844462

[B28] Flier S. N. Tanjore H. Kokkotou E. G. Sugimoto H. Zeisberg M. Kalluri R. Identification of Epithelial to Mesenchymal Transition as a Novel Source of Fibroblasts in Intestinal Fibrosis. J. Biol. Chem. 2010;285:20202–20212. 10.1074/jbc.M110.102012 20363741 PMC2888433

[B29] Franke A. McGovern D. P. Barrett J. C. Wang K. Radford-Smith G. L. Ahmad T. Genome-wide Meta-Analysis Increases to 71 the Number of Confirmed Crohn's Disease Susceptibility Loci. Nat. Genet. 2010;42:1118–1125. 10.1038/ng.717 21102463 PMC3299551

[B30] Galatenko V. V. Galatenko A. V. Samatov T. R. Turchinovich A. A. Shkurnikov M. Y. Makarova J. A. Comprehensive Network of miRNA-Induced Intergenic Interactions and a Biological Role of its Core in Cancer. Sci. Rep. 2018;8:2418. 10.1038/s41598-018-20215-5 29402894 PMC5799291

[B31] Gardner K. E. Allis C. D. Strahl B. D. Operating on Chromatin, a Colorful Language where Context Matters. J. Mol. Biol. 2011;409:36–46. 10.1016/j.jmb.2011.01.040 21272588 PMC3085666

[B32] Garo L. P. Ajay A. K. Fujiwara M. Gabriely G. Raheja R. Kuhn C. MicroRNA-146a Limits Tumorigenic Inflammation in Colorectal Cancer. Nat. Commun. 2021;12:2419. 10.1038/s41467-021-22641-y 33893298 PMC8065171

[B33] Garrido-Mesa J. Rodríguez-Nogales A. Algieri F. Vezza T. Hidalgo-Garcia L. Garrido-Barros M. Immunomodulatory Tetracyclines Shape the Intestinal Inflammatory Response Inducing Mucosal Healing and Resolution. Br. J. Pharmacol. 2018;175:4353–4370. 10.1111/bph.14494 30184260 PMC6240124

[B34] Gasparetto M. Payne F. Nayak K. Kraiczy J. Glemas C. Philip-McKenzie Y. Transcription and DNA Methylation Patterns of Blood-Derived CD8+ T Cells Are Associated with Age and Inflammatory Bowel Disease but Do Not Predict Prognosis. Gastroenterology 2021;160:232–e7. 10.1053/j.gastro.2020.08.017 32814113 PMC7428744

[B35] Glória L. Cravo M. Pinto A. de Sousa L. S. Chaves P. Leitão C. N. DNA Hypomethylation and Proliferative Activity Are Increased in the Rectal Mucosa of Patients with Long-Standing Ulcerative Colitis. Cancer 1996;78:2300–2306. 10.1002/(sici)1097-0142(19961201)78:11<2300:aid-cncr5>3.0.co;2-q 8940998

[B36] Gomez J. A. Wapinski O. L. Yang Y. W. Bureau J. F. Gopinath S. Monack D. M. The NeST Long ncRNA Controls Microbial Susceptibility and Epigenetic Activation of the Interferon-γ Locus. Cell 2013;152:743–754. 10.1016/j.cell.2013.01.015 23415224 PMC3577098

[B37] Gonsky R. Deem R. L. Landers C. J. Derkowski C. A. Berel D. McGovern D. P. Distinct IFNG Methylation in a Subset of Ulcerative Colitis Patients Based on Reactivity to Microbial Antigens. Inflamm. Bowel Dis. 2011;17:171–178. 10.1002/ibd.21352 20848535 PMC3400263

[B38] Gonsky R. Deem R. L. Targan S. R. Distinct Methylation of IFNG in the Gut. J. Interferon Cytokine Res. 2009;29:407–414. 10.1089/jir.2008.0109 19450149 PMC2956574

[B39] Goodrich J. K. Waters J. L. Poole A. C. Sutter J. L. Koren O. Blekhman R. Human Genetics Shape the Gut Microbiome. Cell 2014;159:789–799. 10.1016/j.cell.2014.09.053 25417156 PMC4255478

[B40] Gordon H. Trier Moller F. Andersen V. Harbord M. Heritability in Inflammatory Bowel Disease: from the First Twin Study to Genome-wide Association Studies. Inflamm. Bowel Dis. 2015;21:1428–1434. 10.1097/MIB.0000000000000393 25895112 PMC4450891

[B41] Gruszka R. Zakrzewska M. The Oncogenic Relevance of miR-17-92 Cluster and its Paralogous miR-106b-25 and miR-106a-363 Clusters in Brain Tumors. Int. J. Mol. Sci., 19 2018;19. 10.3390/ijms19030879 PMC5877740 29547527

[B42] Hagihara Y. Yoshimatsu Y. Mikami Y. Takada Y. Mizuno S. Kanai T. Epigenetic Regulation of T Helper Cells and Intestinal Pathogenicity. Semin. Immunopathol 2019;41:379–399. 10.1007/s00281-019-00732-9 30891628

[B43] Harada A. Kimura H. Ohkawa Y. Recent Advances in Single-Cell Epigenomics. Curr. Opin. Struct. Biol. 2021;71:116–122. 10.1016/j.sbi.2021.06.010 34303078

[B44] Harris R. A. Nagy-Szakal D. Mir S. A. Frank E. Szigeti R. Kaplan J. L. DNA Methylation-Associated Colonic Mucosal Immune and Defense Responses in Treatment-Naïve Pediatric Ulcerative Colitis. Epigenetics 2014;9:1131–1137. 10.4161/epi.29446 24937444 PMC4164498

[B45] Häsler R. Feng Z. Bäckdahl L. Spehlmann M. E. Franke A. Teschendorff A. A Functional Methylome Map of Ulcerative Colitis. Genome Res. 2012;22:2130–2137. 10.1101/gr.138347.112 22826509 PMC3483542

[B46] He C. Shi Y. Wu R. Sun M. Fang L. Wu W. miR-301a Promotes Intestinal Mucosal Inflammation through Induction of IL-17A and TNF-α in IBD. Gut 2016;65:1938–1950. 10.1136/gutjnl-2015-309389 26338824

[B47] He C. Yu T. Shi Y. Ma C. Yang W. Fang L. MicroRNA 301A Promotes Intestinal Inflammation and Colitis-Associated Cancer Development by Inhibiting BTG1. Gastroenterology 2017;152:1434–e15. 10.1053/j.gastro.2017.01.049 28193514

[B48] Hou J. Hu X. Chen B. Chen X. Zhao L. Chen Z. miR-155 Targets Est-1 and Induces Ulcerative Colitis via the IL-23/17/6-mediated Th17 Pathway. Pathol. Res. Pract. 2017;213:1289–1295. 10.1016/j.prp.2017.08.001 28888763

[B49] Howell K. J. Kraiczy J. Nayak K. M. Gasparetto M. Ross A. Lee C. DNA Methylation and Transcription Patterns in Intestinal Epithelial Cells from Pediatric Patients with Inflammatory Bowel Diseases Differentiate Disease Subtypes and Associate with Outcome. Gastroenterology 2018;154:585–598. 10.1053/j.gastro.2017.10.007 29031501 PMC6381389

[B50] Issa J. P. Ottaviano Y. L. Celano P. Hamilton S. R. Davidson N. E. Baylin S. B. Methylation of the Oestrogen Receptor CpG Island Links Ageing and Neoplasia in Human colon. Nat. Genet. 1994;7:536–540. 10.1038/ng0894-536 7951326

[B51] James J. P. Riis L. B. Malham M. Høgdall E. Langholz E. Nielsen B. S. MicroRNA Biomarkers in IBD-Differential Diagnosis and Prediction of Colitis-Associated Cancer. Int. J. Mol. Sci., 21 2020;21. 10.3390/ijms21217893 PMC7660644 33114313

[B52] Jeltsch A. Adam S. Dukatz M. Emperle M. Bashtrykov P. Deep Enzymology Studies on DNA Methyltransferases Reveal Novel Connections between Flanking Sequences and Enzyme Activity. J. Mol. Biol. 2021;433:167186. 10.1016/j.jmb.2021.167186 34375615

[B53] Ji Y. Li X. Zhu Y. Li N. Zhang N. Niu M. Faecal microRNA as a Biomarker of the Activity and Prognosis of Inflammatory Bowel Diseases. Biochem. Biophys. Res. Commun. 2018;503:2443–2450. 10.1016/j.bbrc.2018.06.174 29969632

[B54] Johnstone R. W. Histone-deacetylase Inhibitors: Novel Drugs for the Treatment of Cancer. Nat. Rev. Drug Discov. 2002;1:287–299. 10.1038/nrd772 12120280

[B55] Jostins L. Ripke S. Weersma R. K. Duerr R. H. McGovern D. P. Hui K. Y. Host-microbe Interactions Have Shaped the Genetic Architecture of Inflammatory Bowel Disease. Nature 2012;491:119–124. 10.1038/nature11582 23128233 PMC3491803

[B56] Kalla R. Adams A. T. Ventham N. T. Kennedy N. A. White R. Clarke C. Whole Blood Profiling of T-Cell Derived miRNA Allows the Development of Prognostic Models in Inflammatory Bowel Disease. J. Crohns Colitis 2020:14(12):1724–1733. 10.1093/ecco-jcc/jjaa134 32598439

[B57] Karayiannakis A. J. Syrigos K. N. Efstathiou J. Valizadeh A. Noda M. Playford R. J. Expression of Catenins and E-Cadherin during Epithelial Restitution in Inflammatory Bowel Disease. J. Pathol. 1998;185:413–418. 10.1002/(SICI)1096-9896(199808)185:4<413:AID-PATH125>3.0.CO;2-K 9828841

[B58] Kim B. Jeong K. Kim V. N. Genome-wide Mapping of DROSHA Cleavage Sites on Primary MicroRNAs and Noncanonical Substrates. Mol. Cell 2017;66:258–e5. 10.1016/j.molcel.2017.03.013 28431232

[B59] Koizumi K. Alonso S. Miyaki Y. Okada S. Ogura H. Shiiya N. Array-based Identification of Common DNA Methylation Alterations in Ulcerative Colitis. Int. J. Oncol. 2012;40:983–994. 10.3892/ijo.2011.1283 22159500 PMC3584616

[B60] Kosinsky R. L. Zerche M. Kutschat A. P. Nair A. Ye Z. Saul D. RNF20 and RNF40 Regulate Vitamin D Receptor-dependent Signaling in Inflammatory Bowel Disease. Cell Death Differ 2021;28:3161–3175. 10.1038/s41418-021-00808-w 34088983 PMC8563960

[B61] Kraiczy J. Nayak K. Ross A. Raine T. Mak T. N. Gasparetto M. Assessing DNA Methylation in the Developing Human Intestinal Epithelium: Potential Link to Inflammatory Bowel Disease. Mucosal Immunol. 2016;9:647–658. 10.1038/mi.2015.88 26376367 PMC4854977

[B62] Krautkramer K. A. Kreznar J. H. Romano K. A. Vivas E. I. Barrett-Wilt G. A. Rabaglia M. E. Diet-Microbiota Interactions Mediate Global Epigenetic Programming in Multiple Host Tissues. Mol. Cell 2016;64:982–992. 10.1016/j.molcel.2016.10.025 27889451 PMC5227652

[B63] Lee H. S. Impact of Maternal Diet on the Epigenome during In Utero Life and the Developmental Programming of Diseases in Childhood and Adulthood. Nutrients 2015;7:9492–9507. 10.3390/nu7115467 26593940 PMC4663595

[B64] Lee R. C. Feinbaum R. L. Ambros V. The *C. elegans* Heterochronic Gene Lin-4 Encodes Small RNAs with Antisense Complementarity to Lin-14. Cell 1993;75:843–854. 10.1016/0092-8674(93)90529-y 8252621

[B65] Levine A. de Bie C. I. Turner D. Cucchiara S. Sladek M. Murphy M. S. Atypical Disease Phenotypes in Pediatric Ulcerative Colitis: 5-year Analyses of the EUROKIDS Registry. Inflamm. Bowel Dis. 2013;19:370–377. 10.1002/ibd.23013 22570259

[B66] Li J. Zhang J. Guo H. Yang S. Fan W. Ye N. Critical Role of Alternative M2 Skewing in miR-155 Deletion-Mediated Protection of Colitis. Front Immunol. 2018;9:904. 10.3389/fimmu.2018.00904 29774026 PMC5943557

[B67] Lim W. C. Hanauer S. B. Li Y. C. Mechanisms of Disease: Vitamin D and Inflammatory Bowel Disease. Nat. Clin. Pract. Gastroenterol. Hepatol. 2005;2:308–315. 10.1038/ncpgasthep0215 16265284

[B68] Liu M. Rao H. Liu J. Li X. Feng W. Gui L. The Histone Methyltransferase SETD2 Modulates Oxidative Stress to Attenuate Experimental Colitis. Redox Biol. 2021;43:102004. 10.1016/j.redox.2021.102004 34020310 PMC8141928

[B69] Liu S. Rezende R. M. Moreira T. G. Tankou S. K. Cox L. M. Wu M. Oral Administration of miR-30d from Feces of MS Patients Suppresses MS-like Symptoms in Mice by Expanding Akkermansia Muciniphila. Cell Host Microbe 2019;26:779–e8. 10.1016/j.chom.2019.10.008 31784260 PMC6948921

[B70] Lu L. F. Gasteiger G. Yu I. S. Chaudhry A. Hsin J. P. Lu Y. A Single miRNA-mRNA Interaction Affects the Immune Response in a Context- and Cell-type-specific Manner. Immunity 2015;43:52–64. 10.1016/j.immuni.2015.04.022 26163372 PMC4529747

[B71] Lukovac S. Belzer C. Pellis L. Keijser B. J. de Vos W. M. Montijn R. C. Differential Modulation by Akkermansia Muciniphila and Faecalibacterium Prausnitzii of Host Peripheral Lipid Metabolism and Histone Acetylation in Mouse Gut Organoids. mBio, 5 2014;5. 10.1128/mBio.01438-14 PMC4145684 25118238

[B72] Mannino G. Caradonna F. Cruciata I. Lauria A. Perrone A. Gentile C. Melatonin Reduces Inflammatory Response in Human Intestinal Epithelial Cells Stimulated by Interleukin-1β. J. Pineal Res. 2019;67, e12598. 10.1111/jpi.12598 31349378

[B73] McDermott E. Ryan E. J. Tosetto M. Gibson D. Burrage J. Keegan D. DNA Methylation Profiling in Inflammatory Bowel Disease Provides New Insights into Disease Pathogenesis. J. Crohns Colitis 2016;10:77–86. 10.1093/ecco-jcc/jjv176 26419460 PMC5013897

[B74] Mirza A. H. Berthelsen C. H. Seemann S. E. Pan X. Frederiksen K. S. Vilien M. Transcriptomic Landscape of lncRNAs in Inflammatory Bowel Disease. Genome Med. 2015;7:39. 10.1186/s13073-015-0162-2 25991924 PMC4437449

[B75] Murugaiyan G. Garo L. P. Weiner H. L. MicroRNA-21, T Helper Lineage and Autoimmunity. Oncotarget 2015;6:9644–9645. 10.18632/oncotarget.3928 25991670 PMC4496385

[B76] Navarro E. Funtikova A. N. Fíto M. Schröder H. Prenatal Nutrition and the Risk of Adult Obesity: Long-Term Effects of Nutrition on Epigenetic Mechanisms Regulating Gene Expression. J. Nutr. Biochem. 2017;39:1–14. 10.1016/j.jnutbio.2016.03.012 27156216

[B77] Neudecker V. Haneklaus M. Jensen O. Khailova L. Masterson J. C. Tye H. Myeloid-derived miR-223 Regulates Intestinal Inflammation via Repression of the NLRP3 Inflammasome. J. Exp. Med. 2017;214:1737–1752. 10.1084/jem.20160462 28487310 PMC5460990

[B78] Nie L. Wu H. J. Hsu J. M. Chang S. S. Labaff A. M. Li C. W. Long Non-coding RNAs: Versatile Master Regulators of Gene Expression and Crucial Players in Cancer. Am. J. Transl Res. 2012;4:127–150. 22611467 PMC3353529

[B79] Nielsen H. M. Tost J. Epigenetic Changes in Inflammatory and Autoimmune Diseases. Subcell Biochem. 2013;61:455–478. 10.1007/978-94-007-4525-4_20 23150263

[B80] Nimmo E. R. Prendergast J. G. Aldhous M. C. Kennedy N. A. Henderson P. Drummond H. E. Genome-wide Methylation Profiling in Crohn's Disease Identifies Altered Epigenetic Regulation of Key Host Defense Mechanisms Including the Th17 Pathway. Inflamm. Bowel Dis. 2012;18:889–899. 10.1002/ibd.21912 22021194

[B81] Noronha A. M. Liang Y. Hetzel J. T. Hasturk H. Kantarci A. Stucchi A. Hyperactivated B Cells in Human Inflammatory Bowel Disease. J. Leukoc. Biol. 2009;86:1007–1016. 10.1189/jlb.0309203 19589946 PMC2796625

[B82] Oikonomopoulos A. Polytarchou C. Joshi S. Hommes D. W. Iliopoulos D. Identification of Circulating MicroRNA Signatures in Crohn's Disease Using the Nanostring nCounter Technology. Inflamm. Bowel Dis. 2016;22:2063–2069. 10.1097/MIB.0000000000000883 27542126

[B83] Orr B. A. Haffner M. C. Nelson W. G. Yegnasubramanian S. Eberhart C. G. Decreased 5-hydroxymethylcytosine Is Associated with Neural Progenitor Phenotype in normal Brain and Shorter Survival in Malignant Glioma. PLoS One 2012;7, e41036. 10.1371/journal.pone.0041036 22829908 PMC3400598

[B84] Padua D. Mahurkar-Joshi S. Law I. K. Polytarchou C. Vu J. P. Pisegna J. R. A Long Noncoding RNA Signature for Ulcerative Colitis Identifies IFNG-AS1 as an Enhancer of Inflammation. Am. J. Physiol. Gastrointest. Liver Physiol. 2016;311:G446–G457. 10.1152/ajpgi.00212.2016 27492330 PMC5076004

[B85] Pan W. H. Sommer F. Falk-Paulsen M. Ulas T. Best P. Fazio A. Exposure to the Gut Microbiota Drives Distinct Methylome and Transcriptome Changes in Intestinal Epithelial Cells during Postnatal Development. Genome Med. 2018;10:27. 10.1186/s13073-018-0534-5 29653584 PMC5899322

[B86] Pei X. F. Cao L. L. Huang F. Qiao X. Yu J. Ye H. Role of miR-22 in Intestinal Mucosa Tissues and Peripheral Blood CD4+ T Cells of Inflammatory Bowel Disease. Pathol. Res. Pract. 2018;214:1095–1104. 10.1016/j.prp.2018.04.009 29880327

[B87] Perconti G. Rubino P. Contino F. Bivona S. Bertolazzi G. Tumminello M. RIP-chip Analysis Supports Different Roles for AGO2 and GW182 Proteins in Recruiting and Processing microRNA Targets. BMC Bioinformatics 2019;20:120. 10.1186/s12859-019-2683-y 30999843 PMC6471694

[B88] Quigley E. M. Epigenetics: Filling in the 'heritability gap' and Identifying Gene-Environment Interactions in Ulcerative Colitis. Genome Med. 2012;4:72. 10.1186/gm373 23017099 PMC3580442

[B89] Richards E. J. Inherited Epigenetic Variation-Rrevisiting Soft Inheritance. Nat. Rev. Genet. 2006;7:395–401. 10.1038/nrg1834 16534512

[B90] Rosen M. J. Frey M. R. Washington M. K. Chaturvedi R. Kuhnhein L. A. Matta P. STAT6 Activation in Ulcerative Colitis: a New Target for Prevention of IL-13-induced colon Epithelial Cell Dysfunction. Inflamm. Bowel Dis. 2011;17:2224–2234. 10.1002/ibd.21628 21308881 PMC3120916

[B91] Ryan F. J. Ahern A. M. Fitzgerald R. S. Laserna-Mendieta E. J. Power E. M. Clooney A. G. Colonic Microbiota Is Associated with Inflammation and Host Epigenomic Alterations in Inflammatory Bowel Disease. Nat. Commun. 2020;11:1512. 10.1038/s41467-020-15342-5 32251296 PMC7089947

[B92] Saito S. Kato J. Hiraoka S. Horii J. Suzuki H. Higashi R. DNA Methylation of colon Mucosa in Ulcerative Colitis Patients: Correlation with Inflammatory Status. Inflamm. Bowel Dis. 2011;17:1955–1965. 10.1002/ibd.21573 21830274

[B93] Sanchez H. N. Moroney J. B. Gan H. Shen T. Im J. L. Li T. B Cell-Intrinsic Epigenetic Modulation of Antibody Responses by Dietary Fiber-Derived Short-Chain Fatty Acids. Nat. Commun. 2020;11:60. 10.1038/s41467-019-13603-6 31896754 PMC6940392

[B94] Scarpa M. Stylianou E. Epigenetics: Concepts and Relevance to IBD Pathogenesis. Inflamm. Bowel Dis. 2012;18:1982–1996. 10.1002/ibd.22934 22407855

[B95] Schönauen K. Le N. von Arnim U. Schulz C. Malfertheiner P. Link A. Circulating and Fecal microRNAs as Biomarkers for Inflammatory Bowel Diseases. Inflamm. Bowel Dis. 2018;24:1547–1557. 10.1093/ibd/izy046 29668922

[B96] Scharl M. Weber A. Fürst A. Farkas S. Jehle E. Pesch T. Potential Role for SNAIL Family Transcription Factors in the Etiology of Crohn's Disease-Associated Fistulae. Inflamm. Bowel Dis. 2011;17:1907–1916. 10.1002/ibd.21555 21830269

[B97] Singh U. P. Murphy A. E. Enos R. T. Shamran H. A. Singh N. P. Guan H. miR-155 Deficiency Protects Mice from Experimental Colitis by Reducing T Helper Type 1/type 17 Responses. Immunology 2014;143:478–489. 10.1111/imm.12328 24891206 PMC4212960

[B98] Somineni H. K. Venkateswaran S. Kilaru V. Marigorta U. M. Mo A. Okou D. T. Blood-Derived DNA Methylation Signatures of Crohn's Disease and Severity of Intestinal Inflammation. Gastroenterology 2019;156:2254–e3. 10.1053/j.gastro.2019.01.270 30779925 PMC6529254

[B99] Stadhouders R. Lubberts E. Hendriks R. W. A Cellular and Molecular View of T Helper 17 Cell Plasticity in Autoimmunity. J. Autoimmun. 2018;87:1–15. 10.1016/j.jaut.2017.12.007 29275836

[B100] Tahara T. Shibata T. Nakamura M. Yamashita H. Yoshioka D. Okubo M. Promoter Methylation of Protease-Activated Receptor (PAR2) Is Associated with Severe Clinical Phenotypes of Ulcerative Colitis (UC). Clin. Exp. Med. 2009;9:125–130. 10.1007/s10238-008-0025-x 19184329

[B101] Tahara T. Shibata T. Nakamura M. Yamashita H. Yoshioka D. Okubo M. Effect of MDR1 Gene Promoter Methylation in Patients with Ulcerative Colitis. Int. J. Mol. Med. 2009;23:521–527. 10.3892/ijmm_00000160 19288029

[B102] Tahara T. Shibata T. Okubo M. Ishizuka T. Nakamura M. Nagasaka M. DNA Methylation Status of Epithelial-Mesenchymal Transition (EMT)--related Genes Is Associated with Severe Clinical Phenotypes in Ulcerative Colitis (UC). PLoS One 2014;9, e107947. 10.1371/journal.pone.0107947 25303049 PMC4193736

[B103] Treviño L. S. Dong J. Kaushal A. Katz T. A. Jangid R. K. Robertson M. J. Epigenome Environment Interactions Accelerate Epigenomic Aging and Unlock Metabolically Restricted Epigenetic Reprogramming in Adulthood. Nat. Commun. 2020;11:2316. 10.1038/s41467-020-15847-z 32385268 PMC7210260

[B104] Tsaprouni L. G. Ito K. Powell J. J. Adcock I. M. Punchard N. Differential Patterns of Histone Acetylation in Inflammatory Bowel Diseases. J. Inflamm. (Lond) 2011;8:1. 10.1186/1476-9255-8-1 21272292 PMC3040698

[B105] Ueno A. Ghosh A. Hung D. Li J. Jijon H. Th17 Plasticity and its Changes Associated with Inflammatory Bowel Disease. World J. Gastroenterol. 2015;21:12283–12295. 10.3748/wjg.v21.i43.12283 26604637 PMC4649113

[B106] Urich M. A. Nery J. R. Lister R. Schmitz R. J. Ecker J. R. MethylC-seq Library Preparation for Base-Resolution Whole-Genome Bisulfite Sequencing. Nat. Protoc. 2015;10:475–483. 10.1038/nprot.2014.114 25692984 PMC4465251

[B107] Ventham N. T. Kennedy N. A. Adams A. T. Kalla R. Heath S. O'Leary K. R. Integrative Epigenome-wide Analysis Demonstrates that DNA Methylation May Mediate Genetic Risk in Inflammatory Bowel Disease. Nat. Commun. 2016;7:13507. 10.1038/ncomms13507 27886173 PMC5133631

[B108] von Knethen A. Heinicke U. Weigert A. Zacharowski K. Brüne B. Histone Deacetylation Inhibitors as Modulators of Regulatory T Cells. Int. J. Mol. Sci., 21 2020;21. 10.3390/ijms21072356 PMC7177531 32235291

[B109] Wang H. Chao K. Ng S. C. Bai A. H. Yu Q. Yu J. Pro-inflammatory miR-223 Mediates the Cross-Talk between the IL23 Pathway and the Intestinal Barrier in Inflammatory Bowel Disease. Genome Biol. 2016;17:58. 10.1186/s13059-016-0901-8 27029486 PMC4815271

[B110] Wang J. P. Dong L. N. Wang M. Guo J. Zhao Y. Q. MiR-146a Regulates the Development of Ulcerative Colitis via Mediating the TLR4/MyD88/NF-Κb Signaling Pathway. Eur. Rev. Med. Pharmacol. Sci. 2019;23:2151–2157. 10.26355/eurrev_201903_17260 30915760

[B111] Wang S. Hou Y. Chen W. Wang J. Xie W. Zhang X. KIF9‑AS1, LINC01272 and DIO3OS lncRNAs as Novel Biomarkers for Inflammatory Bowel Disease. Mol. Med. Rep. 2018;17:2195–2202. 10.3892/mmr.2017.8118 29207070 PMC5783463

[B112] Wang S. Wan X. Ruan Q. The MicroRNA-21 in Autoimmune Diseases. Int. J. Mol. Sci., 17 2016;17. 10.3390/ijms17060864 PMC4926398 27271606

[B113] Wang Z. Yuan X. Jiao N. Zhu H. Zhang Y. Tong J. CDH13 and FLBN3 Gene Methylation Are Associated with Poor Prognosis in Colorectal Cancer. Pathol. Oncol. Res. 2012;18:263–270. 10.1007/s12253-011-9437-0 21796503

[B114] Wawrzyniak M. Scharl M. Genetics and Epigenetics of Inflammatory Bowel Disease. Swiss Med. Wkly 2018;148, w14671. 10.4414/smw.2018.14671 30378641

[B115] Wei M. Gao X. Liu L. Li Z. Wan Z. Dong Y. Visceral Adipose Tissue Derived Exosomes Exacerbate Colitis Severity via Pro-inflammatory MiRNAs in High Fat Diet Fed Mice. ACS Nano 2020;14:5099–5110. 10.1021/acsnano.0c01860 32275391

[B116] White C. A. Pone E. J. Lam T. Tat C. Hayama K. L. Li G. Histone Deacetylase Inhibitors Upregulate B Cell microRNAs that Silence AID and Blimp-1 Expression for Epigenetic Modulation of Antibody and Autoantibody Responses. J. Immunol. 2014;193:5933–5950. 10.4049/jimmunol.1401702 25392531 PMC4258531

[B117] Wu F. Guo N. J. Tian H. Marohn M. Gearhart S. Bayless T. M. Peripheral Blood microRNAs Distinguish Active Ulcerative Colitis and Crohn's Disease. Inflamm. Bowel Dis. 2011;17:241–250. 10.1002/ibd.21450 20812331 PMC2998576

[B118] Xu M. Zuo D. Liu X. Fan H. Chen Q. Deng S. MiR-155 Contributes to Th17 Cells Differentiation in Dextran Sulfate Sodium (DSS)-induced Colitis Mice via Jarid2. Biochem. Biophys. Res. Commun. 2017;488:6–14. 10.1016/j.bbrc.2017.04.143 28461115

[B119] Xu X. M. Zhang H. J. miRNAs as New Molecular Insights into Inflammatory Bowel Disease: Crucial Regulators in Autoimmunity and Inflammation. World J. Gastroenterol. 2016;22:2206–2218. 10.3748/wjg.v22.i7.2206 26900285 PMC4734997

[B120] Yang H. Y. Barbi J. Wu C. Y. Zheng Y. Vignali P. D. Wu X. MicroRNA-17 Modulates Regulatory T Cell Function by Targeting Co-regulators of the Foxp3 Transcription Factor. Immunity 2016;45:83–93. 10.1016/j.immuni.2016.06.022 27438767 PMC4957244

[B121] Yang Y. Guan J. Shaikh A. S. Liang Y. Sun L. Wang M. Histone Acetyltransferase Mof Affects the Progression of DSS-Induced Colitis. Cell Physiol Biochem 2018;47:2159–2169. 10.1159/000491527 29975939

[B122] Yu T. Guo F. Yu Y. Sun T. Ma D. Han J. Fusobacterium Nucleatum Promotes Chemoresistance to Colorectal Cancer by Modulating Autophagy. Cell 2017;170:548–e16. 10.1016/j.cell.2017.07.008 28753429 PMC5767127

[B123] Zahm A. M. Thayu M. Hand N. J. Horner A. Leonard M. B. Friedman J. R. Circulating microRNA Is a Biomarker of Pediatric Crohn Disease. J. Pediatr. Gastroenterol. Nutr. 2011;53:26–33. 10.1097/MPG.0b013e31822200cc 21546856 PMC3807879

[B124] Zhang M. Zhou L. Zhang S. Yang Y. Xu L. Hua Z. Bifidobacterium Longum Affects the Methylation Level of Forkhead Box P3 Promoter in 2, 4, 6-trinitrobenzenesulphonic Acid Induced Colitis in Rats. Microb. Pathog. 2017;110:426–430. 10.1016/j.micpath.2017.07.029 28733028

[B125] Zhang T. Cooper S. Brockdorff N. The Interplay of Histone Modifications - Writers that Read. EMBO Rep. 2015;16:1467–1481. 10.15252/embr.201540945 26474904 PMC4641500

[B126] Zhernakova A. van Diemen C. C. Wijmenga C. Detecting Shared Pathogenesis from the Shared Genetics of Immune-Related Diseases. Nat. Rev. Genet. 2009;10:43–55. 10.1038/nrg2489 19092835

[B127] Zhou H. Xiao J. Wu N. Liu C. Xu J. Liu F. MicroRNA-223 Regulates the Differentiation and Function of Intestinal Dendritic Cells and Macrophages by Targeting C/EBPβ. Cell Rep 2015;13:1149–1160. 10.1016/j.celrep.2015.09.073 26526992

